# CaMKIV Signaling Is Not Essential for the Maintenance of Intrinsic or Synaptic Properties in Mouse Visual Cortex

**DOI:** 10.1523/ENEURO.0135-21.2021

**Published:** 2021-07-02

**Authors:** Nicholas F. Trojanowski, Gina G. Turrigiano

**Affiliations:** Department of Biology, Brandeis University, Waltham, MA 02454

**Keywords:** CaMKIV, firing rate homeostasis, homeostatic plasticity, intrinsic excitability, synaptic strength

## Abstract

Pyramidal neurons in rodent visual cortex homeostatically maintain their firing rates *in vivo* within a target range. In young cultured rat cortical neurons, Ca^2+^/calmodulin-dependent kinase IV (CaMKIV) signaling jointly regulates excitatory synaptic strength and intrinsic excitability to allow neurons to maintain their target firing rate. However, the role of CaMKIV signaling in regulating synaptic strength and intrinsic excitability *in vivo* has not been tested. Here, we show that in pyramidal neurons in visual cortex of juvenile male and female mice, CaMKIV signaling is not essential for the maintenance of basal synaptic or intrinsic properties. Neither CaMKIV conditional knock-down nor viral expression of dominant negative CaMKIV (dnCaMKIV) *in vivo* disrupts the intrinsic excitability or synaptic input strength of pyramidal neurons in primary visual cortex (V1), and CaMKIV signaling is not required for the increase in intrinsic excitability seen following monocular deprivation (MD). Viral expression of constitutively active CaMKIV (caCaMKIV) *in vivo* causes a complex disruption of the neuronal input/output function but does not affect synaptic input strength. Taken together, these results demonstrate that although augmented *in vivo* CaMKIV signaling can alter neuronal excitability, either endogenous CaMKIV signaling is dispensable for maintenance of excitability, or impaired CaMKIV signaling is robustly compensated.

## Significance Statement

Homeostatic regulation of neuronal activity is essential for stable network function. However, little is known about the mechanisms that coordinate intrinsic and synaptic properties to regulate activity levels *in vivo*. Ca^2+^/calmodulin-dependent kinase IV (CaMKIV) signaling regulates synaptic strength and intrinsic excitability in cultured neurons, but its role in these processes *in vivo* has never been tested. Here, we show that while CaMKIV signaling can modulate intrinsic excitability, it does not play an essential role in maintenance of intrinsic or synaptic properties *in vivo*.

## Introduction

Homeostatic plasticity bidirectionally constrains neuronal activity to a target range by adjusting the relationship between neuronal inputs and firing rate (Turrigiano, 2012; Davis, 2013). Across a variety of vertebrate and invertebrate systems, homeostatic compensatory mechanisms overcome prolonged experimentally-induced changes in activity to return neuronal or network output to a baseline level (Chub and O’Donovan, 1998; Thoby-Brisson and Simmers, 1998; Burrone et al., 2002; Sakurai and Katz, 2009; Harley et al., 2015). The two most studied forms of homeostatic plasticity in the mammalian cortex are synaptic scaling and plasticity of intrinsic excitability (Turrigiano et al., 1998; Desai et al., 1999). During synaptic scaling, the strengths of all synapses onto a neuron are multiplicatively renormalized (Turrigiano and Nelson, 2004), while plasticity of intrinsic excitability modulates the excitability of the neuron as a whole primarily by modulating the abundance and biophysical properties of ion channels (Zhang and Linden, 2003). In rodent primary visual cortex (V1), monocular deprivation (MD) during the critical period first causes a decrease in firing rate in excitatory neurons responding to the deprived eye, followed by a homeostatic return to each neuron’s own original firing rate (Hengen et al., 2016), demonstrating that each excitatory neuron has its own firing rate set point. Synaptic scaling and plasticity of intrinsic excitability can work cooperatively to homeostatically adjust the relationship between neuronal input and output, suggesting that they are jointly regulated (Lambo and Turrigiano, 2013). However, the signaling pathways that allow neurons to coordinate intrinsic and synaptic parameters to maintain these cell-specific firing rate set points *in vivo* are not well understood.

In cultured neurons, Ca^2+^/calmodulin-dependent kinase (CaMK) signaling modulates baseline firing rate by jointly regulating synaptic scaling and intrinsic plasticity (Ibata et al., 2008; Goold and Nicoll, 2010; Joseph and Turrigiano, 2017). CaMK signaling links membrane Ca^2+^ flux to gene transcription, and the rate limiting step in this pathway, phosphorylation of the transcription factor CREB by CaMKIV, is constrained by the amount of CaMKIV in each neuron (Ma et al., 2014; Cohen et al., 2016). Decreasing CaMKIV signaling *in vitro*, mimicking decreased membrane Ca^2+^ entry due to decreased activity, causes synaptic upscaling and increased intrinsic excitability, while increasing CaMKIV signaling has opposing effects. The net result of these coordinated changes in miniature excitatory postsynaptic current (mEPSC) amplitude and intrinsic excitability is a shift in the firing rate of these neurons, arguing that CaMKIV signaling is involved in the regulation of firing rate set points in young neurons *in vitro* (Joseph and Turrigiano, 2017). CaMKIV signaling has also been implicated in the homeostatic regulation of the duration of action potentials that can follow prolonged hyperpolarization *in vitro* (Li et al., 2020). However, role of *in vivo* CaMKIV signaling in regulating synaptic strengths and intrinsic excitability in juvenile animals, at a developmental time when homeostatic plasticity is especially prominent, has not been explored.

Here, we tested if *in vivo* CaMKIV regulates mEPSC amplitude and intrinsic excitability. Using conditional CaMKIV knock-down, we found that decreasing CaMKIV protein levels during the critical period did not alter intrinsic excitability or mEPSC amplitude or frequency in excitatory neurons in layer (L)2/3 or L4 of monocular V1 (V1m), nor did it affect the homeostatic response of pyramidal neurons in V1m or binocular V1 (V1b) to MD. Dominant negative CaMKIV (dnCaMKIV) also failed to alter intrinsic excitability or synaptic properties, while constitutively active CaMKIV (caCaMKIV) caused a complex disruption of intrinsic excitability but did not affect mEPSC frequency or amplitude. These results suggest that CaMKIV signaling is not essential for the regulation of baseline mEPSC properties or intrinsic excitability in the juvenile mouse visual cortex, but do not rule out a role for CaMKIV signaling in acutely modulating intrinsic excitability.

## Materials and Methods

### Mice

All experimental procedures used in this study were approved by the Institutional Animal Care and Use Committee at Brandeis University and followed the guidelines of the National Institutes of Health. Male and female B6.129-Camk4^tm1Gsc^/Ieg mice (EMMA EM:02126, RRID:IMSR_EM:02126) housed in groups were used for all experiments except where noted. In those cases, B6.129-Camk4^tm1Gsc^/Ieg mice were crossed with B6.129S2-Emx1^tm1(cre)Krj^/J (The Jackson Laboratory stock #005628, RRID:IMSR_JAX:005628) mice. All experiments began between zeitgeber time (ZT)0 and ZT4, and concluded by ZT10.

### Virus construction

AAV9-CamKII-HI-eGFP-Cre-WPRE-SV40 (AV-9-PV2521) and AAV9-CamKII0.4-eGFP-WPRE-rBG (AV-9-PV1917) were purchased from the UPenn Vector Core. AAV9-CaMKII0.4-dnCaMKIV-GFP-3xNLS-WPRE and AAV9-CaMKII0.4-caCaMKIV-GFP-3xNLS-WPRE were constructed by replacing the CMV promotor in pEGFP-CaMKIV-CA-Nuc and pEGFP-CaMKIV-DN-Nuc (Schmitt et al., 2004; Wayman et al., 2004) with a 0.4 kb CaMKII promoter fragment (Prakash et al., 2012), then ligating the constructs into an AAV9-MCS backbone (gift from Yasuyuki Shima and Sacha Nelson).

### Virus injection

Stereotactic injection at postnatal day (P)15 or P16 was performed by anesthetizing mice using a ketamine/xylazine/acepromazine mixture, then injecting 200 nl of AAV (diluted 1:10 from titer) from a glass micropipette pulled to a fine point. Injection coordinates (0.5 mm rostral from lambda, 2.5 mm lateral from midline) were scaled to the measured lambda-bregma distance.

### Lid sutures

Lid sutures were performed on P22 or P23. Animals were anesthetized with a ketamine/xylazine/acepromazine mixture, then the right eyelid was trimmed to promote fusion of the top and bottom eyelids during suturing. Eyelids were then sutured together with four 6–0 nylon sutures. Suture integrity was confirmed daily; only animals with intact sutures were used for experiments. For all MD experiments, animals that were anesthetized but not subject to lid suturing (sham) were used as controls.

### Acute slice preparation

At the ages indicated for each experiment (ranging from P22 to P29), animals were deeply anesthetized with isoflurane, then rapidly decapitated. The brain was rapidly removed, then the region containing V1 was cut into 300-μm-thick coronal sections on a vibratome in carbogenated (95% O_2_, 5% CO_2_) standard artificial CSF (ACSF; 126 mm NaCl, 25 mm NaHCO_3_, 3 mm KCl, 2 mm CaCl_2_, 2 mm MgSO_4_, 1 mm NaH_2_PO_4_, and 0.5 mm Na-ascorbate, osmolarity adjusted to 310 mOsm with dextrose, pH 7.35). After slicing, slices were immediately transferred to a 34°C chamber filled with a continuously carbogenated choline-based solution (110 mm choline-Cl, 25 mm NaHCO_3_, 11.6 mm Na-ascorbate, 7 mm MgCl_2_, 3.1 mm Na-pyruvate, 2.5 mm KCl, 1.25 mm NaH_2_PO_4_, and 0.5 mm CaCl_2_, adjusted to 310 mOsm with dextrose, pH 7.35) for 5 min, then transferred back to warm standard ACSF for 30 min (Ting et al., 2014). Slices were then moved to room temperature. Electrophysiological recordings were performed within 6 h of slicing.

### Electrophysiology

In acute slices, V1m and V1b were identified by the shape and morphology of the white matter. Pyramidal neurons were targeted and identified by their teardrop shaped soma and apical dendrite, and their morphology and layer were confirmed *post hoc* by biocytin fills. Borosilicate recording electrodes with tip resistance between 4 and 8 MΩ were pulled on a Sutter P-97 micropipette puller. All recordings were performed on submerged slices continuously perfused with carbogenated 34°C standard ACSF with blockers as indicated below. Neurons were visualized on an Olympus BX51QWI upright epiflouresence microscope with a 10× air (0.13 NA) and 40× water immersion objectives (0.8 NA) with infrared differential interference contrast (DIC) optics and an infrared CCD camera. Data were low-pass filtered at 6 kHz and acquired at 10 kHz with a Multiclamp 700B amplifier and a CV-7b headstage (Molecular Devices). Data were acquired using WaveSurfer (v0.953, Janelia Research Campus), and were analyzed using custom MATLAB scripts. Measurements were not adjusted for the liquid junction potential.

#### Intrinsic excitability measurements

To probe intrinsic excitability, whole cell recordings were performed on pyramidal neurons in current clamp in standard ACSF containing picrotoxin (PTX), 6,7-dinitroquinoxaline-2,3-dione (DNQX), and (2R)-amino-5-phosphonovaleric acid (APV) to block GABA, AMPA, and NMDA receptors, using a K-gluconate-based internal solution containing 100 mm K-gluconate, 10 mm KCl, 10 mm HEPES, 5.37 mm biocytin, 10 mm Na_2_-phosphocreatine, 4 mm Mg-ATP, and 0.3 mm Na-GTP, with sucrose added to bring osmolarity to 295 mOsm and KOH added to bring pH to 7.35 in pipettes with resistance between 4 and 8 mΩ. To maintain resting membrane potential at −70 mV, a small dc bias current was injected. One-second-long current injections of increasing amplitude, ranging from −100 pA to 400 pA in intervals of 20 pA, were delivered to each neuron. Neurons were excluded from analysis if they displayed Rs > 25 MΩ, Vm > −50 mV, or Rin < 80 MΩ. Initial instantaneous firing rate was calculated by taking the reciprocal of the interspike interval between the first two action potentials. Mean instantaneous firing rate was calculated by averaging the reciprocal of the interspike intervals between all action potentials. Action potential threshold was determined by the membrane voltage when the derivative of the voltage first surpassed 20 V/s. Latency was defined as the time to first spike following current injection. Rheobase was defined as the injected current necessary to stimulate an action potential within 500 ms. Action potential half-width was defined as the width of the action potential at the voltage halfway between the peak voltage and the threshold voltage.

#### mEPSC recordings

For spontaneous mEPSC recordings, neurons were voltage clamped to −70 mV in standard ACSF containing tetrodotoxin (TTX) (0.2 μm), APV (50 μm), and PTX (25 μm) to block action potentials, NMDA receptors, and GABA receptors using a Cs-methanesufonate-based internal solution containing 115 mm Cs-methanesulfonate, 10 mm HEPES, 10 mm BAPTA.4Cs, 5.37 mm biocytin, 2 mm QX-314 Cl, 1.5 mm MgCl_2_, 1 mm EGTA, 10 mm Na_2_-phosphocreatine, 4 mm ATP-Mg, and 0.3 mm GTP-Na, with sucrose added to bring osmolarity to 295 mOsm and CsOH added to bring pH to 7.35, in pipettes with resistance between 4 and 8 mΩ. 30 s traces were obtained every minute for 5 min. mEPSC event inclusion criteria included rise times <2 ms and amplitudes >6 pA. Neurons were excluded from analysis if they displayed Rs > 20 MΩ, Vm > −50 mV, or Rin < 80 MΩ.

### Immunohistochemistry

Following transcardial perfusion with 4% paraformaldehyde (PFA) in 0.01 m PBS, brains were postfixed overnight in chilled perfusion solution before being transferred to 0.01 m PBS. 50-μm coronal slices were cut the following day on a vibratome, then washed three times and stored in 0.01 m PBS with 0.05% NaN_3_ at 4°C until staining.

Slices were rinsed three times in 0.01 m PBS, then incubated in the primary antibody solution overnight (0.3% Triton X-100, 0.05% NaN_3_, 1% BSA, and primary antibody in 0.01 m PBS). The following day, slices were rinsed three times in 0.01 m PBS, then incubated in the secondary antibody solution for 2 h (1% BSA and secondary antibody in 0.01 m PBS). Slices were then mounted on a glass slide with an anti-fade medium (Fluoromount-G), coverslipped, and allowed to dry overnight. Images were taken on a Leica SP5 confocal microscope. Image stacks with 488-, 543-, and 647-nm lasers were obtained using a 20× objective with an optical section height (*z*-step) of ∼2 μm. Images were subsequently analyzed in ImageJ.

We used the following antibodies:

anti-CaMKIV (1:1000, Santa Cruz Biotechnology sc-136249, RRID:AB_2275109),

anti-CaMKIV (1:1000, Sigma C2851, RRID:AB_258821),

anti-CaMKIV (1:1000, BD Biosciences 610275, RRID:AB_397670),

anti-pCaMKIV (1:200, Santa Cruz Biotechnology sc-28443-R, RRID:AB_2068399),

anti-VGlut2 (1:10000, Millipore AB2251, RRID:AB_1587626),

Goat anti-Mouse IgG Alexa Fluor 555 (1:300, Thermo Fisher A-21424, RRID:AB_141780),

Goat anti-Rabbit IgG Alexa Fluor 488 (1:300, Thermo Fisher A-11034, RRID:AB_2576217),

Goat anti-Guinea Pig IgG Alexa Fluor 647 (1:300, Thermo Fisher A-21450, RRID:AB_2735091).

When using the polyclonal phospho-CaMKIV (T196) antibody (sc-28443-R, Santa Cruz Biotechnology), we did not observe any difference in staining between wild-type and conditional CaMKIV knock-down mice. This suggests that the Santa Cruz Biotechnology sc-28443-R antibody, when used for immunostaining, is not specific for pCaMKIV. We attempted to immunolabel phospho-CaMKIV with antibodies obtained from multiple other sources with no success.

### Statistical analysis

Data analysis was performed using custom MATLAB scripts or GraphPad Prism. The number of animals and neurons for each experiment, as well as statistical tests used, are stated in each figure legend. Individual data points indicate measurements from one neuron. Horizontal bars and connected points (in F–I curves) represent means, error bars represent SEM unless otherwise noted. Data were tested for normality using a Shapiro–Wilk normality test, and compared using a *t* test (for normally distributed data), Mann–Whitney *U* test (non-normally distributed data), or two-way ANOVA (series data).

## Results

### CaMKIV is expressed in excitatory neurons in mouse V1

CaMKIV is a Ca^2+^-dependent kinase with many targets, including the transcription factor CREB and the splicing factor Nova-2 (Cruzalegui and Means, 1993; Lonze and Ginty, 2002; Li et al., 2020), that has been implicated in synaptic scaling and plasticity of intrinsic excitability in neocortical pyramidal neurons *in vitro* (Joseph and Turrigiano, 2017). However, its role in these processes *in vivo* has not yet been tested.

**Figure 1.**
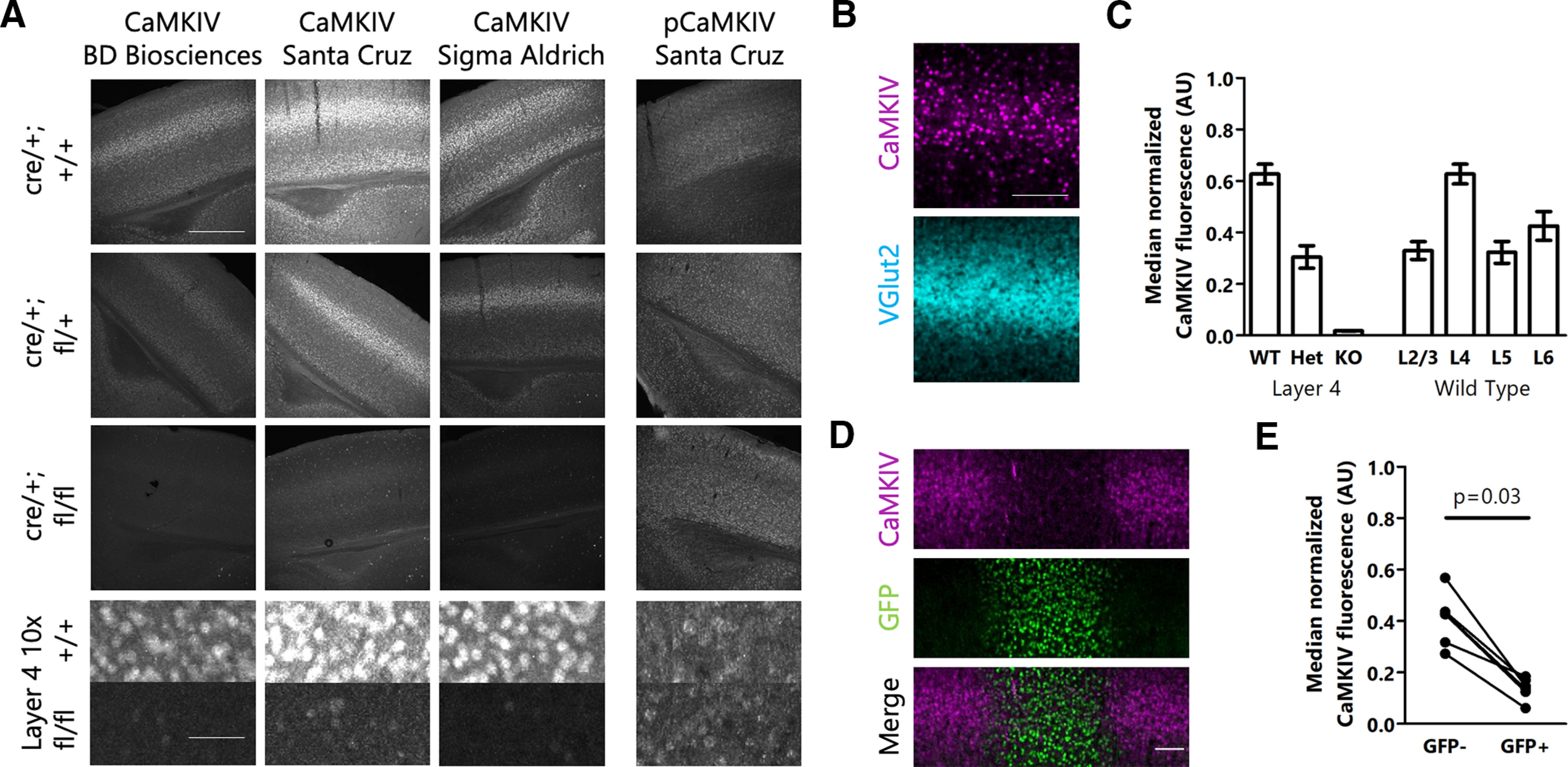
CaMKIV is expressed in excitatory neurons, and at higher levels in L4 of mouse V1. ***A***, anti-CaMKIV and anti-phospho-CaMKIV staining in mouse V1. Each column represents a different antibody, and each row represents a different genotype. Scale bars: 500 μm (top three rows) and 50 μm (bottom row). ***B***, Expression of CaMKIV and VGlut2. Scale bar: 100 μm. ***C***, left, Quantification of CaMKIV expression in mice with 2, 1, or 0 copies of CaMKIV. Right, Quantification of CaMKIV expression in different layers in mice with two copies of CaMKIV. ***D***, Immunofluorescence of CaMKIV and GFP 7 d after Cre-GFP injection into flox-CaMKIV mice. ***E***, Quantification of CaMKIV expression in GFP-negative (GFP–) and GFP-positive (GFP+) neurons 7 d after Cre-GFP injection into flox-CaMKIV mice; *p* value calculated by Wilcoxon matched pairs test.

Before manipulating CaMKIV signaling, we first determined its expression pattern in mouse visual cortex. Using three different primary antibodies, we found that CaMKIV is expressed in a layer-specific manner in mouse visual cortex. Consistent with published RNA-seq data (Hrvatin et al., 2018), we found robust CaMKIV expression in all layers of V1, with highest expression levels in L4 as indicated by its similar expression pattern to that of VGlut2, which labels the thalamocortical afferents that project to L4 (Fig. 1*A*, top row, *B*,*C*). To confirm the specificity of each of these antibodies, and to identify the neuronal types that express CaMKIV in visual cortex *in vivo*, we selectively removed CaMKIV from pyramidal neurons by crossing a flox-CaMKIV mouse (Casanova et al., 2002) with an EMX1-Cre mouse, which expresses Cre exclusively in pyramidal neurons (Gorski et al., 2002). In mice in which both copies of the CaMKIV gene were constitutively removed in pyramidal neurons, we saw a dramatic decrease in CaMKIV expression in all layers regardless of which antibody we used (Fig. 1*A*). We also looked at CaMKIV expression in animals with only one copy of the CaMKIV gene present in excitatory neurons, and found that CaMKIV levels were decreased by about half (Fig. 1*A*). This indicates that these antibodies are specific for CaMKIV and that CaMKIV is primarily expressed in excitatory neurons, consistent with its expression pattern *in vitro* (Cohen et al., 2016; Ma et al., 2014). In contrast, with a commonly used polyclonal phospho-CaMKIV (T196) antibody (sc-28443-R, Santa Cruz Biotechnology) we detected no qualitative difference in labeling between the mice with two copies of the CaMKIV gene and those with zero, arguing that this phospho-CaMKIV antibody is not specific for phospho-CaMKIV (Fig. 1*A*). Attempts to use other phospho-CaMKIV antibodies on tissue slices were unsuccessful.

### CaMKIV signaling *in vivo* is not required for the maintenance of intrinsic excitability or mEPSC amplitude or frequency

Next, we sought to test whether *in vivo* CaMKIV signaling is required for the maintenance of basal excitatory synaptic properties or intrinsic excitability during the rodent visual system critical period. In cultured hippocampal neurons, CaMKIV protein level is rate-limiting in the pathway that links membrane depolarization to CREB phosphorylation, suggesting that altering CaMKIV protein levels should trigger a homeostatic response that can be revealed by measuring basal synaptic and intrinsic properties (Cohen et al., 2016). To avoid compensation that might be arise following constitutive CaMKIV knock-out, we used a viral approach to reduce CaMKIV protein levels with temporal and spatial specificity. We found that 7 d after we injected a Cre-GFP virus (driven by the CaMKII promotor fragment to restrict expression to excitatory neurons) into mouse visual cortex, we saw a robust decrease in CaMKIV protein levels (median decrease of 70%; Fig. 1*D*,*E*).

**Figure 2.**
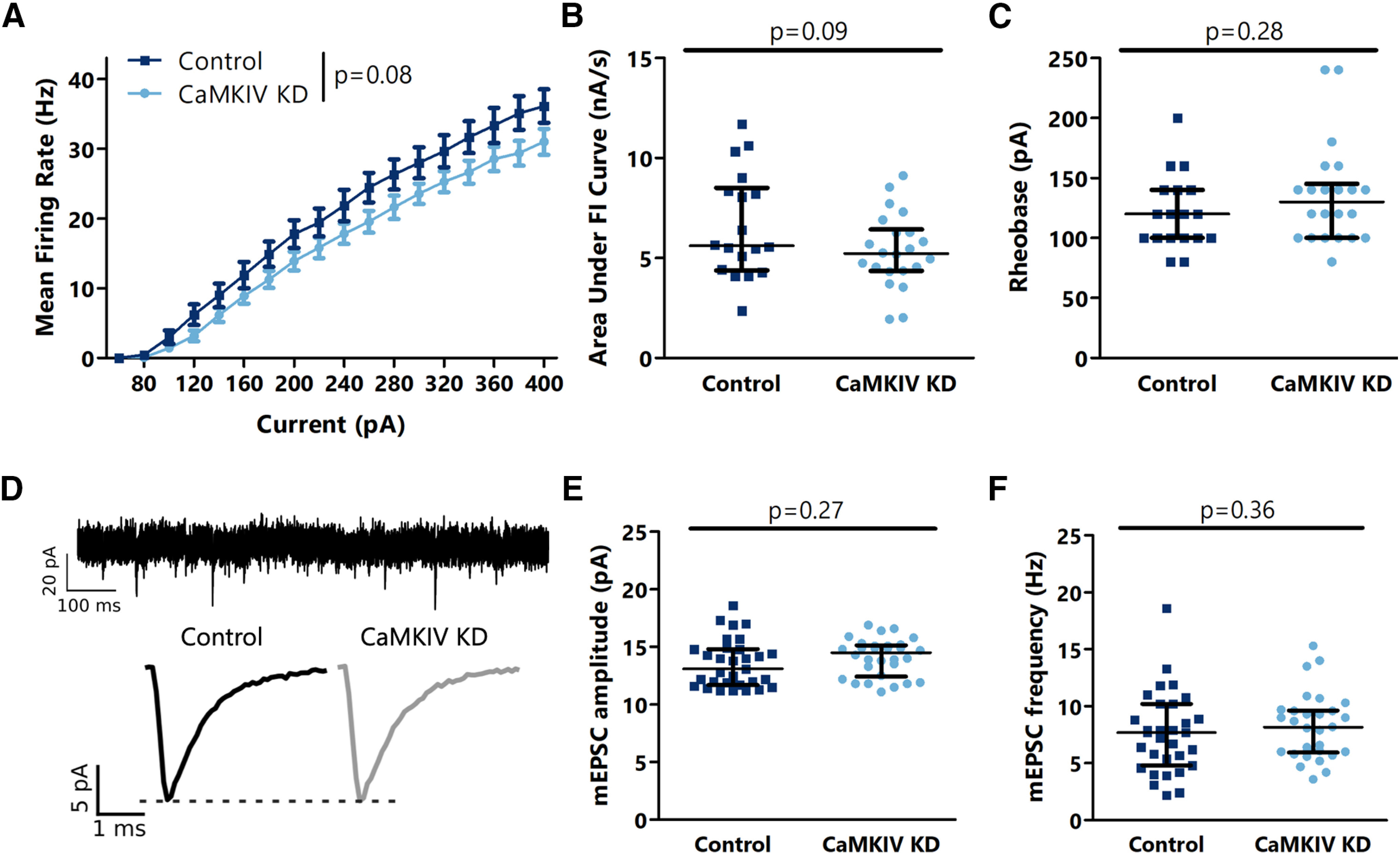
CaMKIV signaling does not negatively regulate intrinsic excitability or mEPSC amplitude or frequency in L2/3 pyramidal neurons in V1m. ***A***, Firing rate-current (F–I) curves for L2/3 control and AAV-mediated CaMKIV knock-down neurons. ***B***, Quantification of the area under the F–I curves from ***A***. ***C***, Comparison of rheobase current between L2/3 control and CaMKIV knock-down neurons. ***D***, top, Representative mEPSC trace from L2/3 control neuron. Bottom, Average mEPSC traces for L2/3 control and CaMKIV knock-down neurons. ***E***, Comparison of mEPSC amplitude between L2/3 control and CaMKIV knock-down neurons. ***F***, Comparison of mEPSC frequency between L2/3 control and CaMKIV knock-down neurons. ***A–C***, *n* = 18–22 neurons from 3 animals per condition. ***D–F***, *n* = 30–31 neurons from 4 animals per condition; *p* values calculated by two-way ANOVA in ***A***, *t* test in ***B***, ***E***, and Mann–Whitney *U* test in ***C***, ***F***. For details, see statistical table (Table 1).

Since robust synaptic scaling and plasticity of intrinsic excitability have both been observed in L2/3 pyramidal neurons during the critical period in V1m (Lambo and Turrigiano, 2013), we injected our Cre-GFP into V1m at P15 and measured the intrinsic excitability and mEPSC amplitude and frequency in pyramidal neurons in infected and uninfected control hemispheres one week later. In contrast to the increase in intrinsic excitability and mEPSC amplitude seen in cultured pyramidal neurons following dnCaMKIV expression (Joseph and Turrigiano, 2017), we saw a small but not statistically significant decrease in intrinsic excitability in neurons lacking CaMKIV (Fig. 2*A–C*), and no change in either mEPSC amplitude or frequency (Fig. 2*D–F*).

**Table 1:** Statistical table

	Structure	Statistical test	*p* value	CI/ranks
Figure 1				
e	Not normal	Wilcoxon matched pairs	**0.0313**	21, 0
Figure 2				
a	Series data	Two-way RM ANOVA	0.0846	
b	Normal	Unpaired *t* test	0.085	6.623 ± 0.6184 *N* = 18, 5.374 ± 0.3916 *N* = 22
c	Not normal	Mann–Whitney *U* test	0.2836	330, 490
e	Normal	Unpaired *t* test	0.2694	13.55 ± 0.3736 *N* = 31, 14.09 ± 0.2983 *N* = 30
f	Not normal	Mann–Whitney *U* test	0.3633	897.5, 993.5
Figure 3				
a	Series data	Two-way RM ANOVA	0.085	
b	Normal	Unpaired *t* test	0.5139	10.47 ± 0.5924 *N* = 27, 9.922 ± 0.5781 *N* = 30
c	Not normal	Mann–Whitney *U* test	0.779	765.5, 887.5
e	Normal	Unpaired *t* test	0.7955	14.53 ± 0.3348 *N* = 31, 14.65 ± 0.3131 *N* = 31
f	Normal	Unpaired *t* test	0.1119	9.974 ± 0.5735 *N* = 31, 11.25 ± 0.5429 *N* = 31
Figure 4				
a	Series data	Two-way RM ANOVA	0.3969	
b	Not normal	Mann–Whitney *U* test	0.506	394, 467
c	Not normal	Mann–Whitney *U* test	0.7259	433.5, 427.5
e	Not normal	Mann–Whitney *U* test	0.315	13.95 ± 0.6251 *N* = 17, 12.99 ± 0.7150 *N* = 17
f	Not normal	Mann–Whitney *U* test	0.2658	5.717 ± 0.5063 *N* = 17, 6.576 ± 0.5653 *N* = 17
Figure 5				
a	Series data	Two-way RM ANOVA	0.1488	
a	Series data	Two-way RM ANOVA	0.5697	
b	Normal	Unpaired *t* test	0.1400	5.047 ± 0.3205 *N* = 24, 5.771 ± 0.3480 *N* = 17
b	Normal	Unpaired *t* test	0.5630	4.555 ± 0.4194 *N* = 19, 4.903 ± 0.4186 *N* = 23
c	Normal	Unpaired *t* test	0.0533	156.7 ± 8.982 *N* = 24, 131.8 ± 7.681 *N* = 17
c	Normal	Unpaired *t* test	0.2457	162.1 ± 7.631 *N* = 19, 151.3 ± 5.456 *N* = 23
d	Series data	Two-way RM ANOVA	**0.006**	
d	Series data	Two-way RM ANOVA	**0.0469**	
e	Normal	Unpaired *t* test	**0.0059**	3.974 ± 0.2320 *N* = 21, 5.314 ± 0.3984 *N* = 21
e	Normal	Unpaired *t* test	**0.0442**	4.063 ± 0.3160 *N* = 14, 5.430 ± 0.4976 *N* = 20
f	Not normal	Mann–Whitney *U* test	**0.0072**	556, 347
f	Normal	Unpaired *t* test	**0.0227**	168.6 ± 7.764 *N* = 14, 142.0 ± 7.525 *N* = 20
i	Not normal	Mann–Whitney *U* test	0.7248	455, 406
i	Normal	Unpaired *t* test	0.8161	0.9152 ± 0.03274 *N* = 14, 0.9050 ± 0.02835 *N* = 20
Figure 6				
a	Series data	Two-way RM ANOVA	0.21	
b	Series data	Two-way RM ANOVA	**<0.0001**	
c	Series data	Two-way RM ANOVA	0.662	
c	Series data	Two-way RM ANOVA	**<0.0001**	
d	Not normal	Mann–Whitney *U* test	0.7443	407, 454
d	Normal	Unpaired *t* test	**<0.0001**	2.807 ± 0.09702 *N* = 27, 6.091 ± 0.4462 *N* = 23
e	Normal	Unpaired *t* test	0.4124	−33.44 ± 0.5506 *N* = 20, −32.67 ± 0.7326 *N* = 21
e	Not normal	Mann–Whitney *U* test	**<0.0001**	378, 897
f	Not normal	Mann–Whitney *U* test	0.5929	441, 420
f	Not normal	Mann–Whitney *U* test	**<0.0001**	920, 355
h	Not normal	Mann–Whitney *U* test	0.5401	361, 269
i	Normal	Unpaired *t* test	0.3617	19.53 ± 1.682 *N* = 19, 17.24 ± 1.803 *N* = 16

Boldface indicates *p* < 0.05

**Figure 3.**
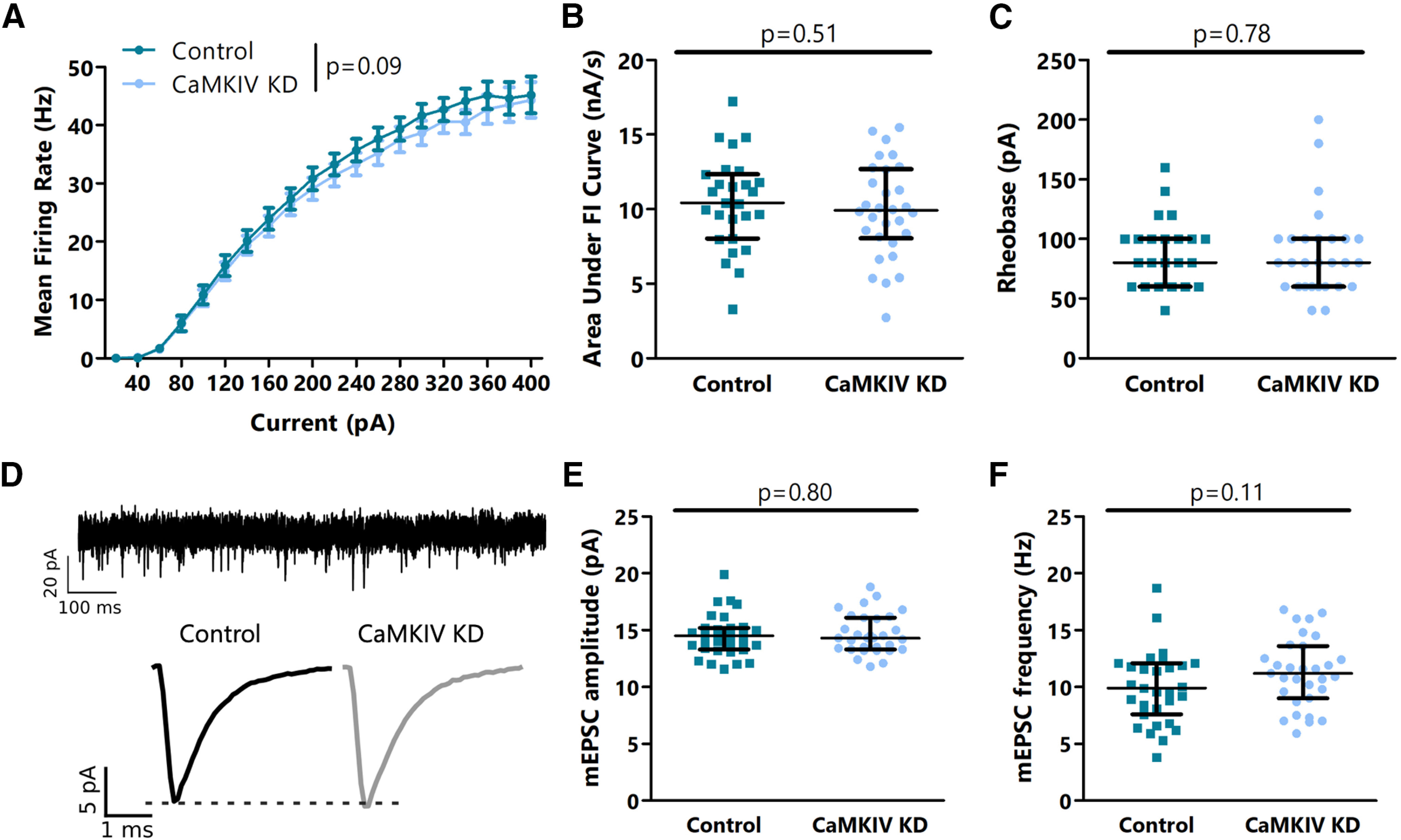
CaMKIV signaling does not negatively regulate intrinsic excitability or mEPSC amplitude or frequency in L4 pyramidal neurons in V1m. ***A***, Firing rate-current (F–I) curves for L4 control and AAV-mediated CaMKIV knock-down neurons. ***B***, Quantification of the area under the F–I curves from ***A***. ***C***, Comparison of rheobase current between L4 control and CaMKIV knock-down neurons. ***D***, top, Representative mEPSC trace from L4 control neuron. Bottom, Average mEPSC traces for L4 control and CaMKIV knock-down neurons. ***E***, Comparison of mEPSC amplitude between L4 control and CaMKIV knock-down neurons. ***F***, Comparison of mEPSC frequency between L4 control and CaMKIV knock-down neurons. ***A–C***, *n* = 27–30 neurons from 4 animals per condition. ***D–F***, *n* = 31 neurons from 4 animals per condition; *p* values calculated by two-way ANOVA in ***A***, *t* test in ***B***, ***E***, ***F***, and Mann–Whitney *U* test in ***C***. For details, see statistical table (Table 1).

To test whether the lack of an effect of decreasing CaMKIV protein levels was layer specific, we next knocked out CaMKIV in L4, where its expression is highest. As in L2/3, we saw a small but not statistically significant decrease in intrinsic excitability following CaMKIV knock-down instead of the expected increase (Fig. 3*A–C*), and no difference in mEPSC amplitude or frequency (Fig. 3*D–F*).

**Figure 4.**
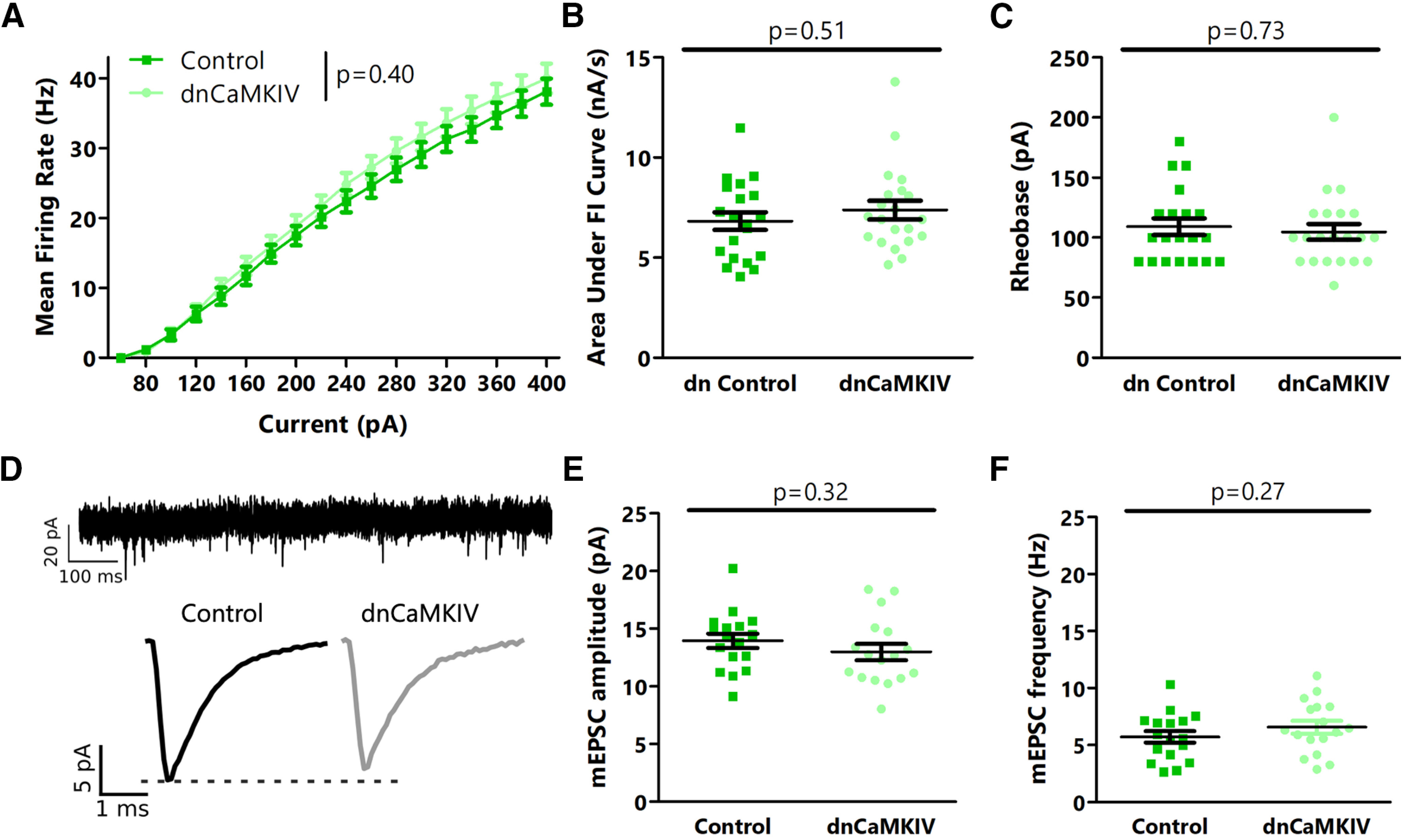
dnCaMKIV signaling does not negatively regulate intrinsic excitability, or mEPSC amplitude or frequency in L2/3 pyramidal neurons in V1m. ***A***, Firing rate-current (F–I) curves for L2/3 control and AAV-mediated dnCaMKIV-expressing neurons. ***B***, Quantification of the area under the F–I curves from ***A***. ***C***, Comparison of rheobase current between L2/3 control and dnCaMKIV. ***D***, top, Representative mEPSC trace from L2/3 control neuron. Bottom, Average mEPSC traces for L2/3 control and dnCaMKIV neurons. ***E***, Comparison of mEPSC amplitude between L2/3 control and dnCaMKIV neurons. ***F***, Comparison of mEPSC frequency between L2/3 control and dnCaMKIV neurons. ***A–C***, *n* = 17 neurons from 3 animals per condition. ***D–F***, *n* = 17 neurons from 3 animals per condition; *p* values calculated by two-way ANOVA in ***A***, *t* test in ***E***, ***F***, and Mann–Whitney *U* test in ***B***, ***C***. For details, see statistical table (Table 1).

Since the *in vitro* data were obtained using dnCaMKIV rather than CaMKIV knock-down, we tested whether our results were because of an unanticipated difference in the effects of CaMKIV knock-down and dnCaMKIV. However, consistent with the our CaMKIV knock-down data, we found that dnCaMKIV expression did not cause an increase in intrinsic excitability (Fig. 4*A–C*) or mEPSC amplitude or frequency (Fig. 4*D–F*). Taken together, our data show that *in vivo* CaMKIV activity is not required for maintaining baseline excitability and mEPSC amplitude in L2/3 or L4 pyramidal neurons.

### CaMKIV signaling is not required for plasticity of intrinsic excitability in V1m or V1b

Although CaMKIV is not required for regulating baseline synaptic and intrinsic properties, it is possible that it is important for homeostatic plasticity in response to persistent changes in input. The intrinsic excitability of excitatory neurons in V1b increases following 6 d of MD (Lambo and Turrigiano, 2013; Moore et al., 2018), but it is not known which molecular pathways regulate this change in intrinsic excitability. The effects of 6 d of MD on intrinsic excitability in V1m are also unknown. To determine whether CaMKIV signaling is required for plasticity of intrinsic excitability we knocked out CaMKIV in V1m or V1b in flox-CaMKIV mice, then sutured one eyelid shut just before the beginning of the critical period (P21) and measured intrinsic excitability 6 d later (Lambo and Turrigiano, 2013).

**Figure 5.**
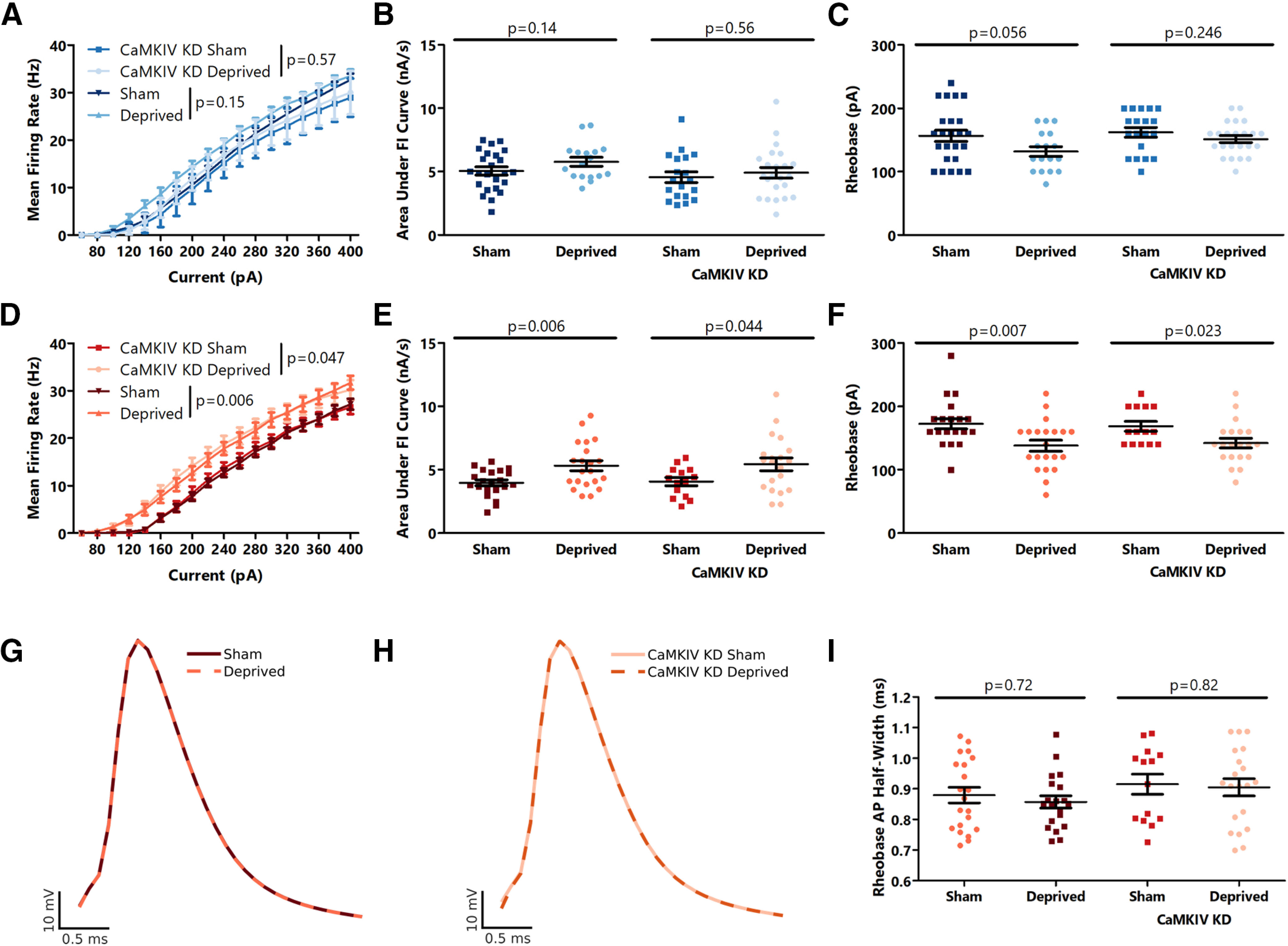
CaMKIV is not required for plasticity of intrinsic excitability in V1m or V1b following a 6-d MD. ***A***, Firing rate-current (F–I) curves for L2/3 sham and monocularly deprived control and CaMKIV knock-down neurons in V1m. ***B***, Quantification of the area under the F–I curves from ***A***. ***C***, Comparison of rheobase current between L2/3 sham and monocularly deprived control and CaMKIV knock-down neurons in V1m. ***D***, Firing rate-current (F–I) curves for L2/3 sham and monocularly deprived control and CaMKIV knock-down neurons in V1b. ***E***, Quantification of the area under the F–I curves from ***D***. ***F***, Comparison of rheobase current between L2/3 sham and monocularly deprived control and CaMKIV knock-down neurons in V1b. ***G***, Average action potential waveforms for the first action potential measured at rheobase for sham and monocularly deprived neurons in V1b. ***H***, Average action potential waveforms for the first action potential measured at rheobase for sham and monocularly deprived CaMKIV knock-down neurons in V1b. ***I***, Action potential half width at rheobase for sham and monocularly deprived control and CaMKIV knock-down neurons in V1b. All comparisons are to mice of the same genotype that were reared and treated identically, except eyelids were not sutured; ***A–C***. *n* = 17–24 neurons from 3 animals per condition. ***D–I***: *n* = 18 neurons from 3 animals per condition. *p* values calculated by two-way ANOVA in ***A, D***, *t*-test in ***B***, ***C***, ***E***, ***F***, ***I***, and Mann-Whitney *U*-test in ***E***, ***F***, ***I***. For details, see statistical table (Table 1).

In V1m, we found that 6 d of MD did not cause a change in intrinsic excitability (Fig. 5*A–C*). As before (Fig. 2*A–C*), we found that CaMKIV knock-down caused a slight decrease in intrinsic excitability, but the decrease in CaMKIV levels did not alter the lack of effect of 6 d of MD on intrinsic excitability. We observed an increased intrinsic excitability in V1b control animals following 6 d of MD, as expected, but this plasticity was also not affected by CaMKIV knock-down (Fig. 5*D–F*). Thus, *in vivo* CaMKIV signaling is not required for the regulation of intrinsic excitability in L2/3 pyramidal neurons in response to MD.

CaMKIV signaling has also recently been implicated in the homeostatic regulation of the width of rebound action potentials that can follow prolonged hyperpolarization *in vitro* (Li et al., 2020). However, its role in regulating the width of action potentials produced by direct depolarization has never been tested. To determine whether action potential width is homeostatically regulated in pyramidal neurons in visual cortex, we measured the width of action potentials following rheobase current injection in V1b in our sham and monocularly deprived animals described above. We found that although 6 d of MD caused a clear increase in intrinsic excitability in V1b, it did not affect action potential width (Fig. 5*G*,*I*). CaMKIV knock-down also failed to alter the action potential width in sham or deprived mice (Fig. 5*H*,*I*). Together, these results demonstrate that as with intrinsic excitability, *in vivo* CaMKIV signaling does not regulate action potential width in pyramidal neurons in visual cortex.

### Constitutive activation of CaMKIV causes complex changes in intrinsic properties

Our data show that *in vivo* CaMKIV signaling is dispensable for homeostatic regulation of neuronal excitability in rodent visual cortex. However, they do not rule out the possibility that *in vivo* CaMKIV signaling does influence mEPSC amplitude or intrinsic signaling excitability, but its loss is robustly compensated. Acute expression of caCaMKIV in pyramidal neurons in rodent hippocampus induces silent synapses, which can be detected as to an increase in mEPSC frequency, despite the lack of effect of dnCaMKIV on hippocampal mEPSCs (Marie et al., 2005). To determine whether a similar phenomenon occurs in pyramidal neurons in rodent visual cortex, we virally expressed caCaMKIV *in vivo* and measured intrinsic and synaptic properties.

**Figure 6.**
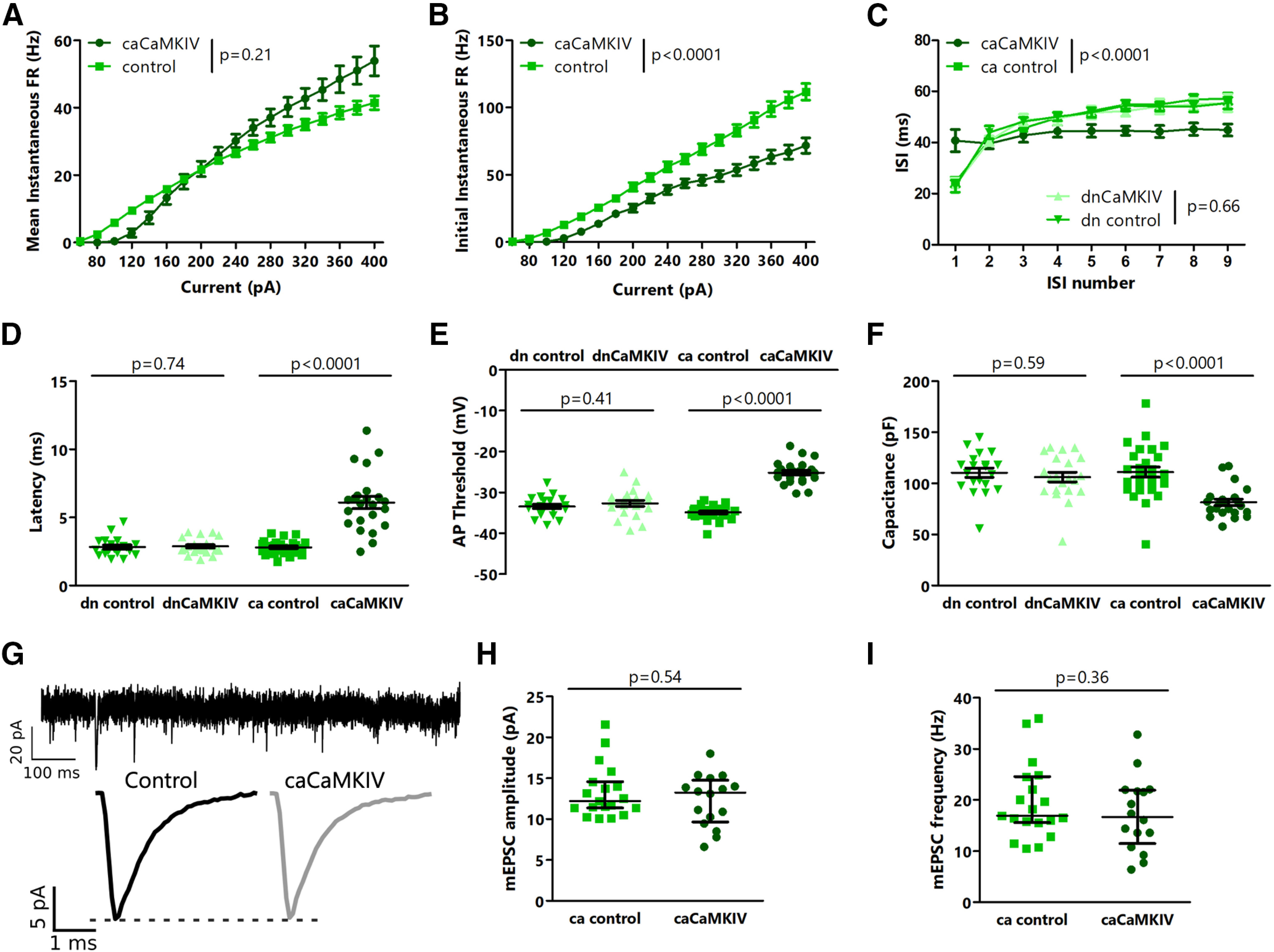
caCaMKIV disrupts intrinsic excitability but does not affect mEPSC amplitude or frequency. ***A***, Mean instantaneous firing rate-current (F–I) curves for L2/3 control and AAV-mediated caCaMKIV-expressing neurons. ***B***, Initial instantaneous firing rate-current (F–I) curves for L2/3 control and AAV-mediated caCaMKIV-expressing neurons. ***C***, Comparison of interspike interval versus spike number for caCaMKIV and dnCaMKIV neurons. ***D***, Comparison of latency to first action potential for caCaMKIV and dnCaMKIV neurons. ***E***, Comparison of action potential threshold for caCaMKIV and dnCaMKIV neurons. ***F***, Comparison of membrane capacitance for caCaMKIV and dnCaMKIV neurons. ***G***, top, Representative mEPSC trace from L2/3 control neuron. Bottom, Average mEPSC traces for L2/3 control and caCaMKIV neurons. ***H***, Comparison of mEPSC amplitude between L2/3 control and caCaMKIV neurons. ***I***, Comparison of mEPSC frequency between L2/3 control and caCaMKIV neurons. ***A–F***, *n* = 18–27 neurons from 3 animals per condition. ***G–I***, *n* = 16–19 neurons from 4 animals per condition; *p* values calculated by two-way ANOVA in ***A–C***, *t* test in ***D***, ***E***, ***I***, and Mann–Whitney *U* test in ***D***, ***E***, ***F***, ***H***. For details, see statistical table (Table 1).

In stark contrast to our observations following dnCaMKIV expression, we found that caCaMKIV, in which the autoinhibitory domain is inactivated (Wayman et al., 2011), caused robust and complex changes in intrinsic excitability. Neurons expressing caCaMKIV showed decreased excitability in response to small amplitude current injections, but with larger current injections their excitability was increased (Fig. 6*A*). Neurons expressing caCaMKIV also showed a lower initial instantaneous firing rate (Fig. 6*B*), less spike frequency adaptation during the spike train (Fig. 6*C*), tended to fire with a delay following depolarization (Fig. 6*D*), and had a higher action potential threshold than control neurons (Fig. 6*E*). They did not show any differences in input resistance, but had an increased resting membrane potential and decreased capacitance (Fig. 6*F*). In contrast, none of these parameters differed between dnCaMKIV and control neurons (Fig. 6*C–F*). These myriad differences following caCaMKIV expression suggest a complex rebalancing of ion channel expression or conductance as a result of constitutive kinase activity.

Finally, we tested whether caCaMKIV also affects synaptic properties by measuring mEPSC amplitude and frequency. As with the CaMKIV knock-down and dnCaMKIV, and in contrast to previous reports from hippocampal pyramidal neurons (Marie et al., 2005), we saw no difference in mEPSC amplitude or frequency between caCaMKIV neurons and control neurons (Fig. 6*G–I*). Taken together, these results demonstrate that constitutive CaMKIV signaling *in vivo* can alter neuronal excitability but not mEPSC frequency or amplitude in cortical pyramidal neurons, but do not support a clear role for CaMKIV signaling in regulating baseline neuronal excitability.

## Discussion

Multiple forms of homeostatic plasticity, most notably synaptic scaling and plasticity of intrinsic excitability, stabilize firing rates of cortical neurons within target ranges. In immature cultured cortical neurons, CaMKIV signaling modulates baseline firing rate by jointly regulating excitatory synaptic strength and intrinsic excitability (Joseph and Turrigiano, 2017), suggesting that it may serve as a master regulator of different forms of homeostatic plasticity. Here, we tested whether *in vivo* CaMKIV signaling similarly regulates baseline synaptic properties and intrinsic excitability in rodent visual cortex, at a time point (the visual system critical period) when neuronal properties and circuits are highly plastic. We found that neither conditional CaMKIV knock-down nor dnCaMKIV mimicked the increase in mEPSC amplitude and intrinsic excitability seen with dnCaMKIV expression in cultured neurons. Similarly, CaMKIV signaling was not necessary for the increase in intrinsic excitability seen in V1b following 6-d MD (Lambo and Turrigiano, 2013; Moore et al., 2018), and neither MD nor CaMKIV knock-down caused homeostatic regulation of action potential width. Expression of caCaMKIV caused a number of changes in intrinsic properties but did not affect mEPSC amplitude or frequency. Taken together, these results suggest that CaMKIV signaling *in vivo* is not essential for homeostatic plasticity in pyramidal neurons in visual cortex, and argue against its role as a general regulator of firing rate set points since it does not regulate baseline mEPSC amplitude or intrinsic excitability. This lack of impact of CaMKIV knock-down on basal properties is consistent with a previous study showing no changes in the levels of pCREB, a downstream CaMKIV effector, across five different brain regions in a constitutive CaMKIV knock-out mouse (Wei et al., 2002).

It is possible that the discrepancies between the effects of CaMKIV manipulation *in vivo* and *in vitro* are because of important experimental differences. Cultured rat cortical neurons were used between 7 and 14 d *in vitro* after being harvested on P0, while our *ex vivo* experiments were performed on mice ages P22 to P29 (Ibata et al., 2008; Joseph and Turrigiano, 2017), so it is possible that the pathways that regulate basal electrophysiological properties and homeostatic plasticity are strongly developmentally regulated, differentially expressed in mice and rats, or both. The duration of CaMKIV manipulation may be significant, as *in vitro* experiments have generally used either short pharmacological manipulations or brief genetic manipulations lasting only 24 h or so (Pratt et al., 2011; Joseph and Turrigiano, 2017) while our approach (conditional knock-down or viral expression *in vivo*) limited us to examining the impact of longer manipulations. It is possible that decreasing CaMKIV signaling for a week or more *in vivo* results in robust compensation. Like CaMKIV, CaMKI is activated by phosphorylation from CaMKK (Selbert et al., 1995) and can itself phosphorylate CREB at ser133 (Ginty et al., 1993), making it a particularly attractive candidate for a compensatory kinase; CaMKK has also been implicated in homeostatic plasticity (Ibata et al., 2008; Goold and Nicoll, 2010; Pratt et al., 2011; Li et al., 2020), making it another attractive candidate. However, acute expression of dnCaMKIV in rodent hippocampal neurons *in vivo* also had no effect on synaptic properties (Marie et al., 2005), consistent with a lack of a role for *in vivo* CaMKIV signaling in regulating baseline excitability.

CaMKIV signaling has recently been implicated in the TTX-induced homeostatic broadening of action potentials caused by rebound following hyperpolarization in cultured hippocampal neurons (Li et al., 2020). In contrast, we found that 6 d of MD, a protocol sufficient to trigger plasticity of intrinsic excitability, did not cause a broadening of action potentials triggered by direct depolarization. Similarly, CaMKIV knock-down did not affect action potential width in sham or monocularly deprived animals. This result suggests that CaMKIV signaling does not regulate action potential width *ex vivo* under physiological conditions, and that regulation of action potential width may not be a prominent feature of homeostatic plasticity of intrinsic excitability in cortical pyramidal neurons.

The function of CaMKIV signaling in cultured neurons may also be distinct from its function *in vivo* in L2/3 or L4. Firing rate set points are homeostatically maintained at the single neuron level *in vivo* (Hengen et al., 2016; Dhawale et al., 2017; Torrado Pacheco et al., 2021), but at the network level in cultured neurons (Slomowitz et al., 2015; Styr et al., 2019), which lack the stereotypical layered structure of intact cortex. This suggests that the specific connectivity of neurons may be critical for their expression of homeostatic plasticity. Since connectivity between neurons across and within layers *in vivo* is influenced by their developmental identity (Cadwell et al., 2019, 2020), a loss of layer-specific identity *in vitro* may lead to aberrant relationships between CaMKIV expression (which is layer-specific *in vivo*), synaptic connectivity, and expression of homeostatic plasticity. It is possible that this disruption causes CaMKIV to assume a role in regulating neuronal excitability *in vitro* that it does not play *in vivo*.

The diverse effects of caCaMKIV expression on various intrinsic synaptic properties likely result from a complex redistribution of the expression of different ionic currents. In particular, the increase in action potential latency along with a decrease in spike frequency adaptation suggest an up-regulation of a transient K^+^ current. The cumulative effects of these changes on the F–I curve of these neurons suggests that they may achieve a greater tendency toward an all or none response, where weak inputs are less likely to elicit a response while strong inputs elicit rapid firing.

However, care must be taken with the interpretation of these data from caCaMKIV, as expression of a constitutively active kinase could lead to off-target effects and overwhelm protein phosphatases. Indeed, it is possible that CaMKIV signaling plays no appreciable role in the maintenance of basal intrinsic excitability and synaptic input strength or homeostatic changes in these parameters in V1 pyramidal neurons *in vivo*, and that the effects of caCaMKIV expression are not physiologically relevant. This does not exclude a potential role for CaMKIV signaling in activity regulation in other excitatory or inhibitory neuron types, brain regions, or developmental stages; indeed, the effects of acute caCaMKIV expression in pyramidal neurons in the hippocampus *in vivo* differ from those seen here in visual cortex (Marie et al., 2005). Regardless of the explanation, our data are consistent with the relatively subtle phenotypes that have been reported in constitutive CaMKIV knock-out mice (Wei et al., 2002; Ko et al., 2006), and suggest that if CaMKIV signaling in the neocortex does modulate network stability *in vivo*, its loss can be robustly compensated.

## Synthesis

Reviewing Editor: Arianna Maffei, SUNY Stony Brook Decisions are customarily a result of the Reviewing Editor and the peer reviewers coming together and discussing their recommendations until a consensus is reached. When revisions are invited, a fact-based synthesis statement explaining their decision and outlining what is needed to prepare a revision will be listed below. The following reviewer(s) agreed to reveal their identity: Lang Wang.

Both reviewers agree that the study presents interesting results that will advance the field. The quality of the data and analysis was found to be very high. However, the reviewers raised some concerns regarding how strongly the reported data support the conclusions presented by the authors that could be adjusted with manuscript revisions and possibly the addition of supporting data. The primary concern has to do with the lack of evidence of knockout efficiency and CaMKIV inactivation following AAV injections used for all the electrophysiological measurements. More details about the experimental design and quantification of CaMKIV KO would help support the authors’ conclusions.

Clarification of effects in V1m and V1b and of the rationale for comparing mEPSCs versus evoked responses would further strengthen the manuscript.

Detailed comments by the reviewers follow below.

Reviewer #1

This manuscript examines the necessity of CaMKIV signaling in the regulation of (1) basal synaptic strength and intrinsic excitability of L2/3 and L4 pyramidal neurons and (2) deprivation-induced homeostatic plasticity in the monocular V1 area during critical period. The data convincingly show that basal synaptic strength, intrinsic excitability and MD-induced increase in intrinsic excitability were not affected by virally-induced CaMKIV manipulations aimed at deletion or inactivation of CaMKIV. These results contradict early studies in primary cortical cultures by Turrigiano lab and in organotypic slice cultures by Nicoll lab. The authors concluded that CaMKIV does not play a role in homeostatic plasticity in the primary visual cortex in vivo.

While the electrophysiological experiments are of high quality and manuscript is clearly written, I am concerned about experimental design that may affect the conclusions:

1. The experimental design for achieving efficient knockout or inactivation of CaMKIV (or any other protein of interest) is questionable. The AAVs were injected at P15-16, Lid suture started at P22-23, slices were prepared 6 days after deprivation at P28-29. It means that the perturbation started already 1 week after virus injection and the recordings were performed 2 weeks after AAV injections. However, typically it takes ∼ 1 month to get significant knockout by AAV-Cre injection or stable AAV expression in the brain in vivo. This is the standard in the field and I am sure that longer periods of virus expression will result in higher levels of knockdown / inactivation.

It is unclear why the authors decided to use genetic knockouts only for antibody quantification, but not for functional analysis. Moreover, they did not present any evidence of the knockout efficiency and CaMKIV inactivation following AAV injections used for all the electrophysiological measurements. The authors should provide sophisticated quantification of pCaMKIV and CaMKIV by western blot and CaMKIV mRNA levels by rtPCR, in addition to histology providing the data on localization of the expressed virus. My prediction is that efficient genetic knockout of CaMKIV (produced by crossing B6.129-Camk4tm1Gsc/Ieg and B6.129S2-Emx1tm1(cre)Krj/J mice as shown in the Fig. 1) BEFORE the start of visual deprivation or before functional measurements in slices may change the results and the conclusions since we don’t know how many molecules of CaMKIV are essential for maintaining the transcriptional regulation and physiological parameters.

2. All the measurements were performed in slices, so the authors should be more accurate with their conclusions about ‘in vitro’ - ‘in vivo’ homeostatic mechanisms. In the same line, monocular deprivation induces reduction in firing rates in the mV1, so the conclusions should be specific for this specific direction of a perturbation.

Minor:

"mEPSC event inclusion criteria included rise times less than 2 seconds” - should be 2 milliseconds.

Reviewer #2

CaMKIV signaling has been reported to regulate excitatory synaptic strength and neuronal intrinsic excitability of cultured neurons in vitro. However, it is not clear whether CaMKIV signaling exerts similar effects in vivo. In the present study, the authors investigated the role of CaMKIV in the regulation of synaptic strength and intrinsic excitability of V1 pyramidal neurons by using CaMKIV conditional KO mice or virus injection to manipulate the expression of CaMKIV in V1. They found either CaMKIV conditional knockout or viral expression of dominant negative CaMKIV did not alter the firing properties and mEPSCs of pyramidal neurons. Furthermore, knocking out CaMKIV did not prevent the change of intrinsic excitability of V1 pyramidal neurons induced by 6-day monocular deprivation. These results suggest that CaMKIV is likely not involved in the regulation of basal synaptic strength and intrinsic excitability, as well as homeostatic plasticity of cortical excitatory neurons.

In general, the manuscript is in a good shape. The experiments are well performed, the data is solid, and the conclusions are clearly illustrated. However, I still have a few concerns/suggestions as followings:

1. To investigate the role of CaMKIV signaling in regulating basal synaptic transmission, the authors recorded mEPSCs of V1 pyramidal neurons. I would strongly argue that it is not appropriate to use miniature PSCs to measure the level of basal synaptic transmission, as miniature PSCs is more suitable to indicate the numbers of readily released neurotransmitter pools and the connectivity of recorded neuron. Thus, evoked EPSCs at different stimulus intensity in control and CaMKIV conditional KO groups need to be acquired to clarify whether CaMKIV signaling participates in the regulation of basal synaptic strength of V1 pyramidal neurons.

2. To test the possibility that CaMKIV signaling is involved in the homeostatic regulation of intrinsic excitability of V1 pyramidal neurons, the authors performed 6-day monocular deprivation during the critical period for visual cortical plasticity. However, the recordings were carried out on neurons in V1b instead of V1m. This could be a reason why the authors did not observe a significant effect of CaMKIV after visual experience manipulation, considering the compensation may be stronger in V1b than in V1m.

3. It is clearly shown that constitutive activation of CaMKIV induced complex changes of neuronal firing properties, such as initial instantaneous firing rate, ISI, latency, capacitance, and action potential threshold. Although these phenotypes are not the main findings of this research, possible mechanisms should still be discussed.

4. The text needs attention:

1) Some abbreviations are shown without full names at their first appearance, e.g. in Page 7 Paragraph 1, ZT0..ZT4..ZT10; in Page 15 Paragraph 2, V1..; in Page 16 Paragraph 2, “mEPSC” should be moved to the place where it is first mentioned;

2) In Page 7 Paragraph 2, the names of viruses are not in the uniform format;

3) All temperature should be spelled with “℃” but not only “C";

4) In Page 9 Paragraph 2, in the description on the gradients of the internal solution, “5.37mM biocytin” is unusually high. Is that correct?

5) Similarly, in Page 12 line 2, it is stated that anti-VGlut2 is 1:10000. Is that correct?

## Author Response

Response to Reviewers

Synthesis Statement for Author:

Both reviewers agree that the study presents interesting results that will advance the field. The quality of the data

and analysis was found to be very high. However, the reviewers raised some concerns regarding how strongly the

reported data support the conclusions presented by the authors that could be adjusted with manuscript revisions and

possibly the addition of supporting data. The primary concern has to do with the lack of evidence of knockout

efficiency and CaMKIV inactivation following AAV injections used for all the electrophysiological measurements.

More details about the experimental design and quantification of CaMKIV KO would help support the authors’

conclusions. Clarification of effects in V1m and V1b and of the rationale for comparing mEPSCs versus evoked

responses would further strengthen the manuscript. Detailed comments by the reviewers follow below.

We thank the Editors for the opportunity to revise this manuscript, and the Reviewers for their

thorough and constructive feedback. To address their concerns, we have added data from

additional experiments and revised the figures and text. The major additions include:

1. Quantification of CaMKIV protein levels following Cre-dependent knockdown. These

data and an example image are included in Figure 1. We have also revised the text to

more accurately reflect that our CaMKIV manipulation is a knockdown, rather than a

complete knockout.

2. Data from monocular deprivation experiments from V1m. The results of these

experiments are consistent with the data from V1b and from non-MD experiments. These

data have been added to Figure 5.

3. A number of textual changes to more accurately present our findings

Other revisions are described below in our point-by-point response.

Reviewer #1

This manuscript examines the necessity of CaMKIV signaling in the regulation of (1) basal synaptic strength and

intrinsic excitability of L2/3 and L4 pyramidal neurons and (2) deprivation-induced homeostatic plasticity in the

monocular V1 area during critical period. The data convincingly show that basal synaptic strength, intrinsic

excitability and MD-induced increase in intrinsic excitability were not affected by virally-induced CaMKIV

manipulations aimed at deletion or inactivation of CaMKIV. These results contradict early studies in primary

cortical cultures by Turrigiano lab and in organotypic slice cultures by Nicoll lab. The authors concluded that

CaMKIV does not play a role in homeostatic plasticity in the primary visual cortex in vivo.

While the electrophysiological experiments are of high quality and manuscript is clearly written, I am concerned

about experimental design that may affect the conclusions:

1. The experimental design for achieving efficient knockout or inactivation of CaMKIV (or any other protein of

interest) is questionable. The AAVs were injected at P15-16, Lid suture started at P22-23, slices were prepared 6

days after deprivation at P28-29. It means that the perturbation started already 1 week after virus injection and the

recordings were performed 2 weeks after AAV injections. However, typically it takes ∼ 1 month to get significant

knockout by AAV-Cre injection or stable AAV expression in the brain in vivo. This is the standard in the field and I

am sure that longer periods of virus expression will result in higher levels of knockdown / inactivation.

It is unclear why the authors decided to use genetic knockouts only for antibody quantification, but not for

functional analysis. Moreover, they did not present any evidence of the knockout efficiency and CaMKIV

inactivation following AAV injections used for all the electrophysiological measurements. The authors should 2

provide sophisticated quantification of pCaMKIV and CaMKIV by western blot and CaMKIV mRNA levels by

rtPCR, in addition to histology providing the data on localization of the expressed virus. My prediction is that

efficient genetic knockout of CaMKIV (produced by crossing B6.129-Camk4tm1Gsc/Ieg and B6.129S2-

Emx1tm1(cre)Krj/J mice as shown in the Fig. 1) BEFORE the start of visual deprivation or before functional

measurements in slices may change the results and the conclusions since we don’t know how many molecules of

CaMKIV are essential for maintaining the transcriptional regulation and physiological parameters.

We thank the reviewer for raising this point, as it is an important point that we did not explain

with sufficient clarity in our original submission. Cohen et al 2016 have shown in cultured

hippocampal neurons that CaMKIV expression is rate-limiting in the signaling pathway that

links membrane Ca2+ entry to CREB phosphorylation, and that even relatively subtle

manipulation of CaMKIV protein level can affect CREB phosphorylation in response to

depolarization. Further, relatively acute (2 day) expression of dominant negative CaMKIV

(which is unlikely to completely block CaMKIV signaling) modulates both mEPSC amplitude

and intrinsic excitability (Joseph and Turrigiano, 2017). We therefore hypothesized that a graded

decrease in CaMKIV protein levels in vivo would trigger homeostatic compensation, as neurons

would perceive this as a decrease in activity. We used a conditional viral approach rather than a

global genetic knockout approach for the majority of these experiments in order to avoid

compensatory effects that might arise when CaMKIV signaling is constitutively abolished from

birth. Based on the previous literature, our thinking was that manipulating CaMKIV protein

levels after network activity has stabilized would disrupt synaptic and intrinsic properties in a

detectable way.

We have now quantified the effects of our viral CaMKIV manipulation and added this to Figure

1. We find a ∼70% decrease in CaMKIV protein 7 days after AAV injection (Our lab routinely

achieves stable AAV-Cre-dependent virus expression, such as fluorescent reporters, by 7 days

after virus expression), which strongly supports our use of this approach.

We have added this information to the results section. We have also changed all references to

this approach from “CaMKIV knockout” to “CaMKIV knockdown” to more accurately reflect

our perturbation.

2. All the measurements were performed in slices, so the authors should be more accurate with their conclusions

about ‘in vitro’ - ‘in vivo’ homeostatic mechanisms. In the same line, monocular deprivation induces reduction in

firing rates in the mV1, so the conclusions should be specific for this specific direction of a perturbation.

Throughout the text, we have made changes to reflect the fact that the CaMKIV manipulations,

but not the electrophysiological recordings, were performed in vivo. We have also clarified that

we are specifically testing for increases in intrinsic excitability following monocular deprivation

Minor:

"mEPSC event inclusion criteria included rise times less than 2 seconds” - should be 2 milliseconds.

Thanks for catching this, we have made this correction

Reviewer #23

CaMKIV signaling has been reported to regulate excitatory synaptic strength and neuronal intrinsic excitability of

cultured neurons in vitro. However, it is not clear whether CaMKIV signaling exerts similar effects in vivo. In the

present study, the authors investigated the role of CaMKIV in the regulation of synaptic strength and intrinsic

excitability of V1 pyramidal neurons by using CaMKIV conditional KO mice or virus injection to manipulate the

expression of CaMKIV in V1. They found either CaMKIV conditional knockout or viral expression of dominant

negative CaMKIV did not alter the firing properties and mEPSCs of pyramidal neurons. Furthermore, knocking out

CaMKIV did not prevent the change of intrinsic excitability of V1 pyramidal neurons induced by 6-day monocular

deprivation. These results suggest that CaMKIV is likely not involved in the regulation of basal synaptic strength

and intrinsic excitability, as well as homeostatic plasticity of cortical excitatory neurons.

In general, the manuscript is in a good shape. The experiments are well performed, the data is solid, and the

conclusions are clearly illustrated. However, I still have a few concerns/suggestions as followings:

1. To investigate the role of CaMKIV signaling in regulating basal synaptic transmission, the authors recorded

mEPSCs of V1 pyramidal neurons. I would strongly argue that it is not appropriate to use miniature PSCs to

measure the level of basal synaptic transmission, as miniature PSCs is more suitable to indicate the numbers of

readily released neurotransmitter pools and the connectivity of recorded neuron. Thus, evoked EPSCs at different

stimulus intensity in control and CaMKIV conditional KO groups need to be acquired to clarify whether CaMKIV

signaling participates in the regulation of basal synaptic strength of V1 pyramidal neurons.

We thank the reviewer for raising this important point. We used the approach of measuring

mEPSCs so that our results would be directly comparable to previous experiments performed in

Joseph and Turrigiano, 2017, where the authors showed a bidirectional change in mEPSC

amplitude following dnCaMKIV or caCaMKIV expression. To more accurately reflect our data,

we have revised the text to make it clear that we are measuring mESPC amplitude and frequency

rather than evoked EPSC amplitude.

2. To test the possibility that CaMKIV signaling is involved in the homeostatic regulation of intrinsic excitability of

V1 pyramidal neurons, the authors performed 6-day monocular deprivation during the critical period for visual

cortical plasticity. However, the recordings were carried out on neurons in V1b instead of V1m. This could be a

reason why the authors did not observe a significant effect of CaMKIV after visual experience manipulation,

considering the compensation may be stronger in V1b than in V1m.

Our motivation for performing the experiments in V1b is that 6 days monocular deprivation is

reported to cause a homeostatic shift in intrinsic excitability in V1b (Lambo and Turrigiano,

2013; Moore et al., 2018), but the same effect has not been demonstrated in V1m. We performed

identical experiments in V1m and found that, interestingly, 6 days of monocular deprivation does

not cause a shift in intrinsic excitability in mouse V1m. We have now added these data to Figure

5.

3. It is clearly shown that constitutive activation of CaMKIV induced complex changes of neuronal firing properties,

such as initial instantaneous firing rate, ISI, latency, capacitance, and action potential threshold. Although these

phenotypes are not the main findings of this research, possible mechanisms should still be discussed.

We have added a discussion of these possible mechanisms to the discussion section. However,

we also caution against a strong interpretation of these data as constitutive expression of a kinase

may lead to off target effects that are not physiologically relevant.

4. The text needs attention:

1) Some abbreviations are shown without full names at their first appearance, e.g. in Page 7 Paragraph 1,

ZT0...ZT4...ZT10; in Page 15 Paragraph 2, V1; in Page 16 Paragraph 2, “mEPSC” should be moved to the place

where it is first mentioned;4

We have spelled out the abbreviations where they first appear

2) In Page 7 Paragraph 2, the names of viruses are not in the uniform format;

We have corrected the formatting of the names of the viruses used in this study.

3) All temperature should be spelled with “degreesC” but not only “C";

We have made this change throughout

4) In Page 9 Paragraph 2, in the description on the gradients of the internal solution, “5.37mM biocytin” is unusually

high. Is that correct?

This is correct. Biocytin concentrations are usually given in percentages (typically 0.2%, which

is the same as 5.37 mM), but we find it more consistent to report all concentrations in molarity.

5) Similarly, in Page 12 line 2, it is stated that anti-VGlut2 is 1:10000. Is that correct?

This is correct
